# Cost-Effectiveness and Quality of Care of a Comprehensive ART Program in Malawi: Erratum

**DOI:** 10.1097/01.md.0000494746.29563.13

**Published:** 2016-08-26

In the article “Cost-Effectiveness and Quality of Care of a Comprehensive ART Program in Malawi”,^1^ which appeared in Volume 95, Issue 21 of *Medicine*, Dr. Lauren Shear Zinner's surname was originally spelled as Zimmer. The article has since been corrected online.
